# Grey Model Optimized by Particle Swarm Optimization for Data Analysis and Application of Multi-Sensors

**DOI:** 10.3390/s18082503

**Published:** 2018-08-01

**Authors:** Chenming Li, Hongmin Gao, Junlin Qiu, Yao Yang, Xiaoyu Qu, Yongchang Wang, Zhuqing Bi

**Affiliations:** College of Computer and Information, Hohai University, Nanjing 211100, China; lichenming55@163.com (C.L.); 15195891396@163.com (J.Q.); rcyyang@hhu.edu.cn (Y.Y.); quxiaoyu@hhu.edu.cn (X.Q.); wangyongchang@hhu.edu.cn (Y.W.); 15195891861@163.com (Z.B.)

**Keywords:** pumping station, temperature change, multivariable grey system prediction, *q* parameters, multi-sensor temperature data

## Abstract

Data on the effective operation of new pumping station is scarce, and the unit structure is complex, as the temperature changes of different parts of the unit are coupled with multiple factors. The multivariable grey system prediction model can effectively predict the multiple parameter change of a nonlinear system model by using a small amount of data, but the value of its *q* parameters greatly influences the prediction accuracy of the model. Therefore, the particle swarm optimization algorithm is used to optimize the *q* parameters and the multi-sensor temperature data of a pumping station unit is processed. Then, the change trends of the temperature data are analyzed and predicted. Comparing the results with the unoptimized multi-variable grey model and the BP neural network prediction method trained under insufficient data conditions, it is proved that the relative error of the multi-variable grey model after optimizing the *q* parameters is smaller.

## 1. Introduction

In power machinery, the analysis and prediction of the temperature changes of multiple sensors from different parts of the equipment are important bases for the evaluation of its running state [1,2]. Pumping stations are the most widely used water facilities. China has more than 40 large and medium pumping stations, all of which urgently require effective assessment of their pumping station operation status. The pump unit of the pumping station is a typical power mechanical device. The structure of a large pumping station is complex. Many factors, such as water flow, cavitation and other hydraulic factors, spindle bending, asymmetrical and other mechanical factors, short circuits of the stator winding, and overcurrent, can affect the temperature changes in various parts of the pump [3,4]. Temperature variation in various parts often occurs due to the complex coupling of these multiple factors [5]. These coupling actions tends to overlap, resulting in different influences and effects on the temperature in different parts. The analysis results show that analyzing and predicting the temperature change of multiple parts captured by multiple sensors on a pump unit is a multivariable and nonlinear problem [6,7], which is a research hotspot and a difficult concept at present [8,9,10].

Traditional forecasting methods mainly include time series models and regression analyses. These methods can positively predict linear and stationary characteristic quantities. Temperature change data captured by multiple sensors in a pumping station is nonlinear and non-stationary, which prevents the traditional prediction methods from achieving good results. At present, data-driven neural network technology has made rapid progress in the field of prediction. Piotrowski et al. [11] used different neural network models to predict and compare river water temperatures. Drevetskyi et al. [12] used the back propagation (BP) neural network to predict urban water consumption. Tang et al. [13] used the improved BP neural network to predict the bearing bush temperature of hydropower units. However, the prediction method based on neural network requires abundant a priori data as input to obtain accurate and generalized trained models.

Pumping station prototypes and actual pumping stations are different because of their different physical conditions. Specifically, the operation characteristics of the same type of pumping stations are different, and the state analysis model of the pumping station cannot be easily transferred. Effective long sequence operation data of new pumping stations’ pump units are scarce, especially fault and other abnormal performance data. Therefore, temperature changes cannot be completely predicted based on neural networks. The multivariable grey model (MGM) (1, *n*) was developed based on grey system theory [14] proposed by Deng (where (1, *n*) represents First order ordinary differential equation with n elements). It is a multidimensional generalization of the single variable grey model (GM) (1, 1) (where (1, 1) represents a first order ordinary differential equation with one element). MGM can describe the different characteristics that affect the operating state of the system from a multidimensional degree, which can overcome the non-stationary signals limitations and effectively analyze and predict multiple correlation eigenvalues of the system under the condition of a small amount of known information. The model is suitable for analyzing and predicting the temperature variation of multiple parts and multiple sensors in pumping stations.

**Figure 1:**
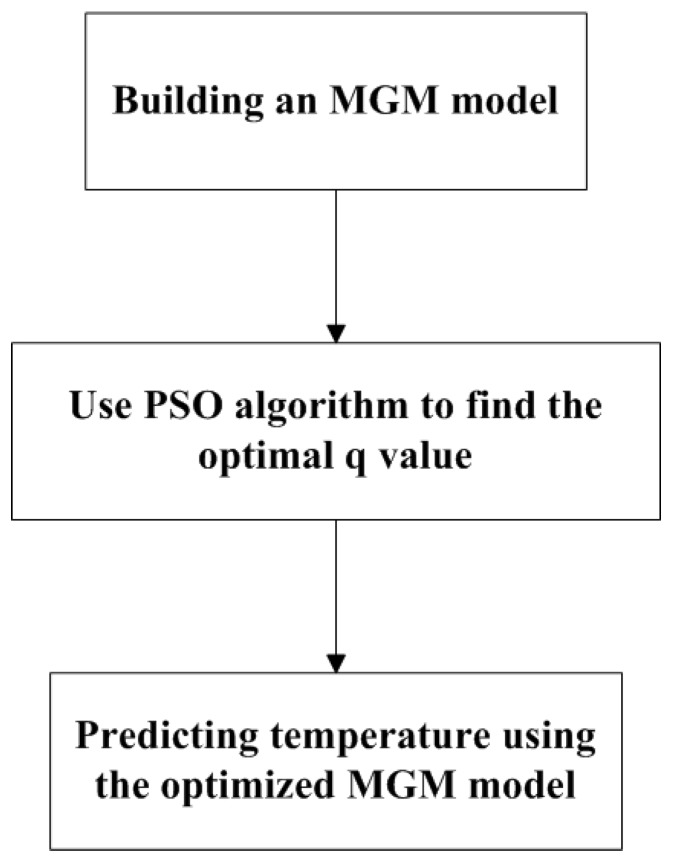
The steps of the PSO algorithms.

Although the MGM (1, *n*) has the capability to predict using a small amount of data, the prediction accuracy is greatly influenced by the parameter *q* values in the model difference expansion. Finding the most suitable *q* value can improve the prediction accuracy of the model, and the search for parameter *q* is an NP-hard problem [15]. The particle swarm optimization (PSO) algorithm is a group intelligent optimization method. Compared with the genetic algorithm, PSO avoids complex operations such as “cross” and “mutation” and has the advantages of rapid convergence and high accuracy [16]. For this reason, an MGM is developed based on the temperature data collected from the upper guide bearing, including the temperature data of the stator winding and of the thrust bearing. Then, the PSO algorithm is used to find its optimal parameter *q* value. Finally, MGM is used to predict the temperature of each part after optimization of the *q* parameters. The procedure is shown in Figure 1. With the same amount of data, the optimized MGM (1, *n*) is compared with the traditional MGM and the prediction model based on the BP neural network. Then, the experimental results are compared. The results show that the MGM after optimization of the *q* parameters is better than the traditional MGM and the BP neural network. The prediction model improved the prediction accuracy by 0.01% and 2.02%, respectively.

## 2. Multivariable Grey Model

MGM (1, *n*, *q*) was developed based on grey system theory. In 1982, Deng published his first paper on the “control of grey system” in the *Journal of System Control and Communication* which received extensive attention. Since grey system theory was developed, an increasing number of scholars have been involved in research attempting to solve many practical problems with the theory and achieving favorable results [17].

For a variable $X_{i}^{\left(0\right)}$, the observed value sequence on the time axis is $X_{i}^{\left(0\right)}=\{x_{i}^{\left(0\right)}(1),x_{i}^{\left(0\right)}(2),\ldots ,x_{i}^{\left(0\right)}(m)\}$. Then, the sequence of the observation values of *n* different variables on the time axis constitutes a data matrix $X^{\left(0\right)}=\{X_{1}^{\left(0\right)},X_{2}^{\left(0\right)},\ldots ,X_{n}^{\left(0\right)}\}$.

Accumulate the sequence of observations for each variable separately, The obtained new data matrix becomes the first-order cumulative generation matrix of the original matrix $X^{\left(0\right)}$, Write it as $X^{\left(1\right)}$, which can be expressed as $X^{\left(1\right)}=\{X_{1}^{\left(1\right)},X_{2}^{\left(1\right)},\ldots ,X_{n}^{\left(1\right)}\}$, where $X_{i}^{\left(1\right)}$ is the first-order cumulative generation sequence of the original data sequence $X_{i}^{\left(0\right)}$, i.e.,:

(1) $$X_{i}^{\left(1\right)}=\{x_{i}^{\left(1\right)}(1),\;x_{i}^{\left(1\right)}(2),\;\ldots ,\;x_{i}^{\left(1\right)}(m)\},$$

(2) $$x_{i}^{\left(1\right)}(j)=\sum_{k=1}^{j}x_{i}^{\left(0\right)}(k),$$

where *i* = 1, 2, …, *m*, and *j* = 1, 2, …, *n*.

Then the matrix form of the MGM (1, *n*) model is as follows:

(3) $$\frac{dX^{\left(1\right)}(t)}{dt}=AX^{\left(1\right)}(t)+B,$$

where $X^{\left(1\right)}(t)=\{x_{1}^{\left(1\right)}(t),x_{2}^{\left(1\right)}(t),\ldots ,x_{n}^{\left(1\right)}(t)\}$, $A={\left(a_{ij}\right)}_{n\times n}$, $B={\left(b_{1},b_{2},\ldots ,b_{n}\right)}^{T}$.

The first-order ordinary differential equation in Equation (3) can obtain its time response formula as follows:

(4) $$X_{}^{\left(1\right)}(t)=e^{A(t-1)}(X_{}^{\left(1\right)}(1)+A^{-1}B)-A^{-1}B,$$

where $e^{At}=I+At+\frac{A^{2}}{2!}t^{2}+\ldots =I+\sum_{k=1}^{\infty }\frac{A^{k}}{k!}t^{k}$, and $X^{\left(1\right)}(1)=\{x_{1}^{\left(1\right)}(1),x_{2}^{\left(1\right)}(1),\ldots ,x_{n}^{\left(1\right)}(1)\}$. Equation (4) can be used to predict the value of the next moment from the value of the previous moment.

Set $A^{\prime }=\left[A,\;B\right]$, the least squares estimate of $a_{j}{}^{T}$ (*j* = 1, 2, …, *n*) is as follows:

(5) $${\hat{a}}_{j}{}^{T}={\left(L^{T}L\right)}^{-1}L^{T}Y_{j},$$

where:

(6) $$L=\left[\begin{array}{lllll} \frac{1}{2}(x_{1}^{1}(2)+x_{1}^{1}(1)) & \frac{1}{2}(x_{2}^{1}(2)+x_{2}^{1}(1)) & \ldots & \frac{1}{2}(x_{n}^{1}(2)+x_{n}^{1}(1)) & 1\\ \frac{1}{2}(x_{1}^{1}(3)+x_{1}^{1}(2)) & \frac{1}{2}(x_{2}^{1}(3)+x_{2}^{1}(2)) & \ldots & \frac{1}{2}(x_{n}^{1}(3)+x_{n}^{1}(2)) & 1\\ \;\;\;\;\vdots & \;\;\;\;\vdots & & \;\;\;\;\vdots & \vdots \\ \frac{1}{2}(x_{1}^{1}(m)+x_{1}^{1}(m-1)) & \frac{1}{2}(x_{2}^{1}(m)+x_{2}^{1}(m-1)) & \ldots & \frac{1}{2}(x_{n}^{1}(m)+x_{n}^{1}(m-1)) & 1 \end{array}\right],$$

and $Y_{j}={\left(x_{i}^{\left(0\right)}(2),x_{i}^{\left(0\right)}(3),\cdots ,x_{i}^{\left(0\right)}(m)\right)}^{T}$.

The forward difference of Formula (3) is divided into $\frac{X_{t+1}^{\left(1\right)}-X_{t}^{\left(1\right)}}{(t+1)-t}=AX_{t}+B$. Collate and obtain the following:

(7) $$X_{t+1}^{\left(1\right)}-AX_{t}^{\left(1\right)}-X_{t}^{\left(1\right)}=B.$$

Equation (3) is divided into $\frac{X_{t}^{\left(1\right)}-X_{t-1}^{\left(1\right)}}{t-(t-1)}=AX_{t}+B$ or $\frac{X_{t+1}^{\left(1\right)}-X_{t}^{\left(1\right)}}{(t+1)-t}=AX_{t+1}+B$ after the backward difference. Collate and obtain the following:

(8) $$\begin{array}{l} \;\;\;\;X_{t}^{\left(1\right)}-AX_{t}^{\left(1\right)}-X_{t-1}^{\left(1\right)}=B.\\ Or\\ \;\;\;\;X_{t+1}^{\left(1\right)}-AX_{t+1}^{\left(1\right)}-X_{t}^{\left(1\right)}=B. \end{array}$$

Equation (7) establishes the MGM (1, *n*, *q*). In special cases, when *q* = 0.5, the model is degenerated into the GM (1, 1) model. When *q* takes a different value *q*_0_, the L in Equation (5) is changed as follows:

(9) $$[\begin{array}{lllll} q_{0}x_{1}^{1}(2)+(1-q_{0})x_{1}^{1}(1) & q_{0}x_{2}^{1}(2)+(1-q_{0})x_{2}^{1}(1)) & \ldots & q_{0}x_{n}^{1}(2)+(1-q_{0})x_{n}^{1}(1)) & 1\\ q_{0}x_{1}^{1}(3)+(1-q_{0})x_{1}^{1}(2)) & q_{0}x_{2}^{1}(3)+(1-q_{0})x_{2}^{1}(2)) & \ldots & q_{0}x_{n}^{1}(3)+(1-q_{0})x_{n}^{1}(2)) & 1\\ \;\;\;\;\;\vdots & \;\;\;\;\;\vdots & & \;\;\;\;\;\vdots & \vdots \\ q_{0}x_{1}^{1}(m)+(1-q_{0})x_{1}^{1}(m-1)) & q_{0}x_{2}^{1}(m)+(1-q_{0})x_{2}^{1}(m-1)) & \ldots & q_{0}x_{n}^{1}(m)+(1-q_{0})x_{n}^{1}(m-1)) & 1 \end{array}].$$

The analysis results show that the different values of *q*_0_ affect the value of L and then affect the fitting and prediction accuracies of MGM (1, *n*, *q*). Therefore, selecting the most suitable *q*_0_ value is necessary to obtain the most accurate model. The optimal value cannot be easily obtained by solving the column equation because a complex nonlinear relationship exists between the value of *q*_0_ and the fitting accuracy of the model. Therefore, a swarm intelligence optimization method, PSO, is introduced to optimize the value of *q*_0_ and improve the fitting accuracy of MGM (1, *n*, *q*).

## 3. PSO-Based *q* Parameter Optimization

PSO was introduced in 1995 by two researchers, Kennedy and Eberhart, who were inspired by the predation behavior of birds. PSO is a typical swarm intelligence optimization method. It is simple in structure, easy to implement, and has rapid convergence and high accuracy. After more than 20 years of development, the theoretical basis of PSO is nearing completion. Many scholars have provided some improvements on the special needs of different optimization problems and have successfully applied these enhancements to the optimization of various practical problems.

Prior to the use of the PSO algorithm to optimize MGM (1, *n*, *q*) models, the following definitions are provided:

**Definition** **1:** *The actual data collected include X = (x_1_, x_2_, …, x_n_). The value of MGM (1, n, q) is X′ = (x_1_′, x_2_′, …, x_n_′). The residual of the model is D = (d_1_, d_2_, …, d_n_) = (x_1_ − x_1_′, x_2_ − x_2_′, …, x_n_ − x_n_′). The relative error is R = (r_1_, r_2_, …, r_n_) = abs(d_1_/x_1_, d_2_/x_2_, …, d_n_/x_n_) × 100%.*

(10) $$v_{i}^{k+1}=\omega v_{i}^{k}+c_{1}\xi ({\tilde{p}}_{i}{}^{k}-\chi_{i}^{k})+c_{2}\eta ({\tilde{g}}^{k}-\chi_{i}^{k}),$$

(11) $$\chi_{i}^{k+1}=\chi_{i}^{k}+v_{i}^{k+1},$$

*where ω ∈ [0, 1] represents inertia weight, c_1_ and c_2_ are the learning factors enabling particles to learn from other excellent individuals,*ξ and η *represent two pseudo random numbers distributed in [0, 1] intervals.* $v_{i}^{k}$ *indicates the speed at which the i particle moves at k times. It represents the inertial effect of the particle’s current velocity on the next movement speed.* ${\tilde{p}}_{i}^{k}$ *represents the optimal position of the individual i particle after k movement.* $c_{1}\xi ({\tilde{p}}_{i}{}^{k}-\chi_{i}^{k})$ *represents the self-cognitive behavior of the particle, and the direction of its next movement, to some extent, refers to the optimal position that it experienced.* ${\tilde{g}}^{k}$ *represents the historical optimal value after the k movement of all particles, and* $c_{2}\eta ({\tilde{g}}^{k}-\chi_{i}^{k})$ *expresses the social learning behavior of the particles and the next shift. The motion direction, to some extent, refers to the optimal position that all particles experienced.* $\chi_{i}^{k}$ *represents the position after the first movement of the i particle. Formula (11) indicates that the position of the particle after the next movement is equal to the current position plus the speed of the next movement.*

The steps of the PSO algorithm when optimizing the MGM (1, *n*, *q*) are as follows:

Step 1: Population initialization, including population *n* and speed *v*.
Step 2: Constructing the objective function *fit* (*q*), as follows:
  (12) $$fit(q(i,k))=\sum_{i=1}^{n}d_{i}{}^{2},$$
  where q(i,k) is the fitness of the *i* particle after the *k* moves. The fitness values of each particle in the population are solved according to the fitness function.
Step 3: Saving the individual historical optimal value ${\tilde{p}}_{i}{}^{k}$ of the particle.
Step 4: Saving the global historical optimal value ${\tilde{g}}^{k}$ of the particle.
Step 5: Step 5: Judging whether the algorithm reaches the prescribed number of iterations. If the condition is satisfied, then the global optimum is outputted; if it is not satisfied, then proceed to Step 6.
Step 6: Step 6: Iteration of updates, according to Formulas (10) and (11).
Step 7: Step 7: Proceed to Step 2.

**Figure 2:**
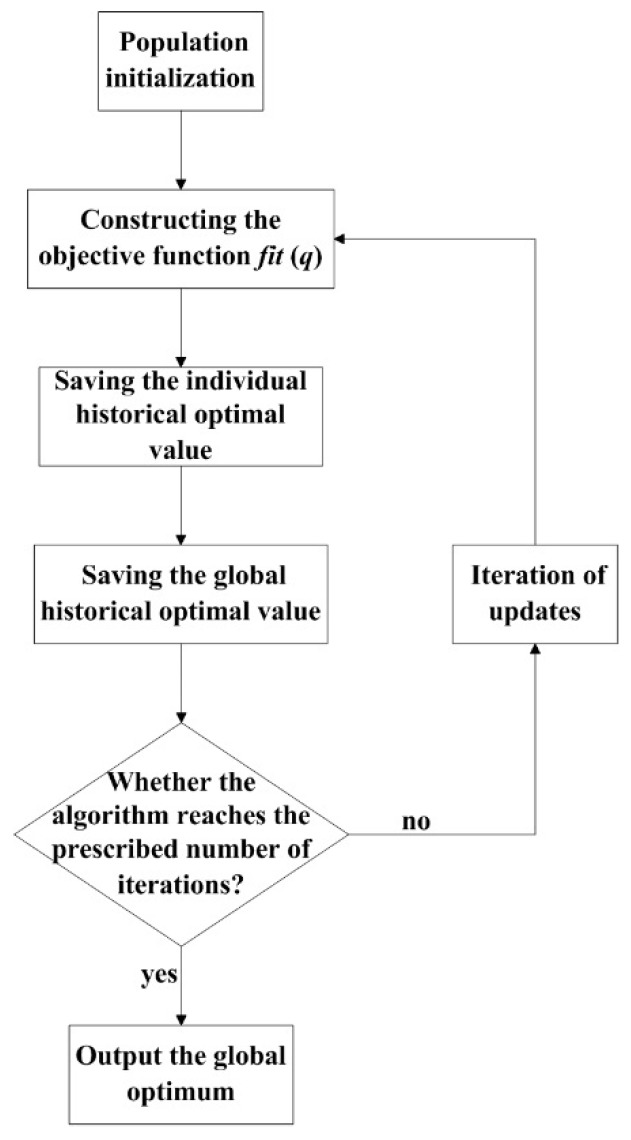
The procedure of MGM model optimized by PSO algorithm.

The detailed procedure is shown in Figure 2.

## 4. Application of PSO to MGM in Temperature Prediction of the Pumping Station Unit

In this paper, the proposed algorithm is applied to the prediction of characteristic quantities in the operation of pump station units of the east line of the south-to-north water transfer project. As the eastern route of the south-to-north water diversion project is just completed, its effective operation time is short, and the accumulated effective data, especially the data and fault data under different working conditions, are very scarce. Currently, the popular data-driven feature volume prediction methods (such as BP neural network) all have high prediction accuracy, but they all need sufficient and effective data as the training basis. When there are few training data, the model trained by this method is often not sufficient, and there are problems such as poor generalization caused by over-fitting and merging. Combined with the application of this project, the experimental results show that when there is less effective running data, it is not good to use the data-driven BP neural network method to predict the feature volume. However, it does not need too much historical data to get a high prediction accuracy by using multi-variable grey model. And the prediction accuracy of the multivariable grey model was further improved after *q* parameters were optimized by particle swarm optimization algorithm.

In the experimental part of this paper, in order to verify the accuracy of the multivariable grey model optimized by particle swarm optimization algorithm in multivariate prediction, the temperature data of guide bearing, stator winding and thrust bearing of unit 3 at a certain period of time during the operation of Hongze Station in the south-to-north water transfer project were collected, four valid digits are retained and the collection time interval is 3 min. The temperature data of these three parts can not only reflect the temperature of each part, but also correlate with each other, the advantages of the optimized multivariable grey model can be demonstrated.

The MGM (1, 3, *q*) is optimized by PSO based on the given data, where *c*_1_ = *c*_2_ = 1.5, maximum iteration number *Maxgen* = 50, population size *Sizepop* = 10, and inertia weight W = 1 − (0.8/*maxgen*) × k. k represents the Kth movement of the particle swarm.

Throughout many experiments, when nine sets of data are obtained, the relative error of MGM (1, 3, *q*) is the smallest. Therefore, the nine sets of data from T1 to T9 are considered the benchmark data in this study. At this time:

(13) $$L=\left[\begin{array}{llll} q_{0}\times 51.87+(1-q_{0})\times 24.24 & q_{0}\times 48.45+(1-q_{0})\times 23.13) & q_{0}\times 43.36+(1-q_{0})\times 21.43 & 1\\ q_{0}\times 81.49+(1-q_{0})\times 51.87) & q_{0}\times 75.79+(1-q_{0})\times 48.45) & q_{0}\times 66.09+(1-q_{0})\times 43.36 & 1\\ \;\;\;\;\vdots & \;\;\;\;\vdots & \;\;\;\vdots \;\;\;\;\;\;\;\vdots & \\ q_{0}\times 246.74+(1-q_{0})\times 212.22) & q_{0}\times 269.77+(1-q_{0})\times 234.46) & q_{0}\times 214.41+(1-q_{0})\times 188.48 & 1 \end{array}\right],$$

and:

(14) $$Y=[\begin{array}{l} 27.63\;\;25.32\;\;21.93\\ 29.62\;\;27.34\;\;22.73\\ 31.31\;\;29.03\;\;23.34\\ 32.32\;\;30.52\;\;23.95\\ 33.30\;\;31.71\;\;24.64\\ 33.80\;\;33.11\;\;25.03\\ 34.52\;\;34.30\;\;25.43\\ 34.81\;\;35.31\;\;25.93 \end{array}].$$

On the basis of numerous PSO calculations, when the parameter *q* = 0.5095, the objective function obtains the best value fit(0.5095) = 0.086. Thus, the model values and predicted values of the MGM (1, 3, 0.5095) and their relative errors to the original data sequence of multi-sensors can be obtained, and two bits are retained. The original data from the experiment, the forecast data from the optimized model, and the relative error between the original data and the forecast data are listed in Table 1.

In Table 1, $x_{1}^{\left(0\right)}(k)$, $x_{2}^{\left(0\right)}(k)$, and $x_{3}^{\left(0\right)}(k)$ represent the original temperature data multi-sensors from the upper guide bearing, stator winding, and the thrust bearing, respectively. ${x^{\prime }}_{1}^{\left(0\right)}(k)$, ${x^{\prime }}_{2}^{\left(0\right)}(k)$, and ${x^{\prime }}_{3}^{\left(0\right)}(k)$ represent the results after fitting the MGM (1, 3, *q*) and the 10th behavioral model predictions. Table 1 shows that the MGM (1, 3, *q*) model has a good fitting effect, with an average relative error of less than 0.26%, and a prediction error of less than 0.99%.

**Figure 3:**
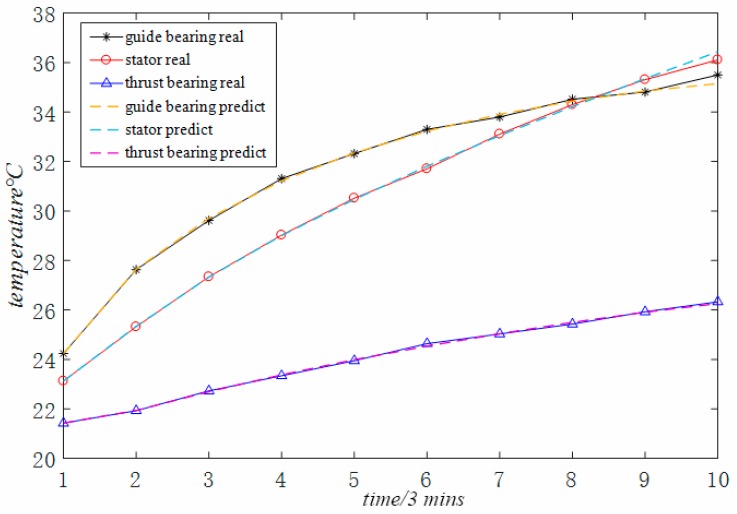
MGM (1, 3, *q*) model prediction effect.

In order to present a more intuitive analysis of the data in the table, the data in the table is transformed into Figure 3. Figure 3 shows the time-varying curve of the original temperature data and the time-varying curve of the data predicted by the optimized grey model proposed in this paper. It can be clearly seen from the figure that the degree of fitting between the original data and the predicted data is relatively high.

## 5. Comparison among Algorithms

**Figure 4:**
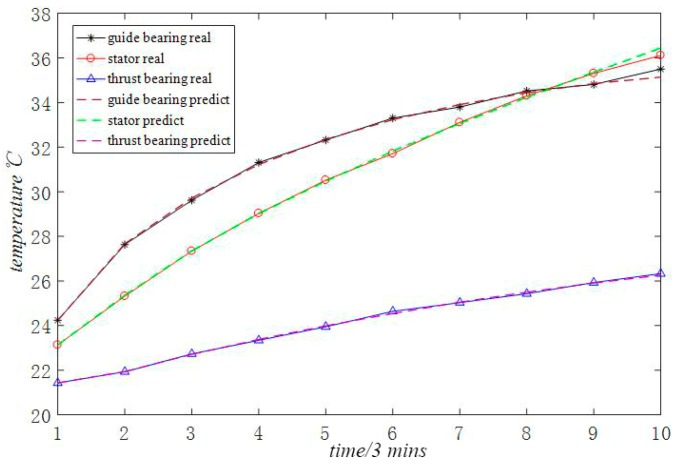
MGM (1, 3) model prediction effect.

**Figure 5:**
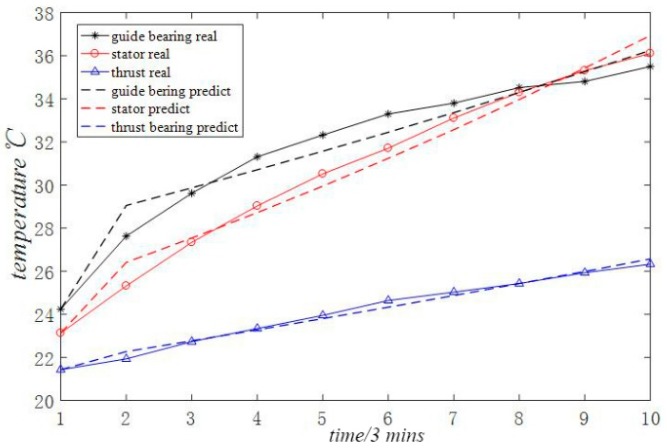
GM (1, 1) model prediction effect.

**Figure 6:**
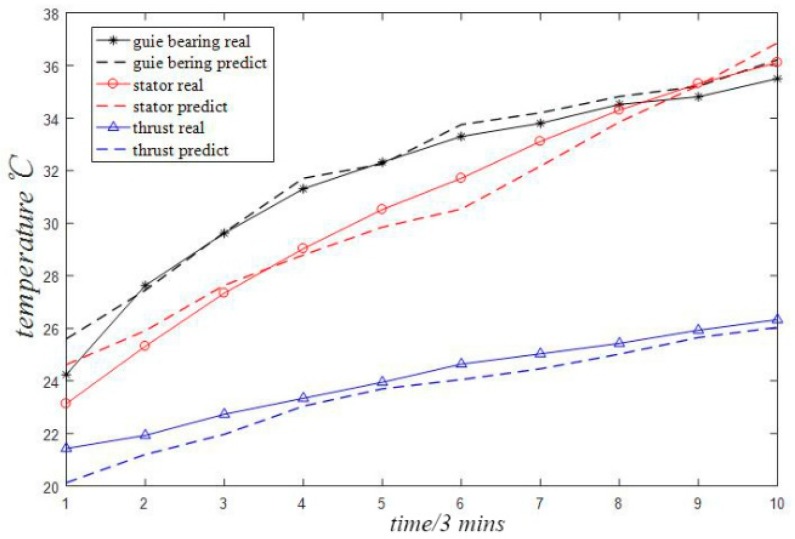
Prediction effect of BP neural network model.

In order to verify the superiority of the proposed algorithm, the proposed multi-variable grey model algorithm optimized by particle swarm optimization is compared with the general multi-variable grey model method without particle swarm optimization, the common single-variable grey model prediction method and the BP neural network method described in [13]. The relative errors between the original data and the predicted data are listed in Table 2, Table 3 and Table 4 as shown in Table 1. From Table 2 to Table 4, it can be found that the prediction accuracy is lower than that of the PSO optimized multi-variable grey model in this paper. Among them, the prediction accuracy of the common single variable grey model shown in Table 3 and the neural network method shown in Table 4 is relatively low. Accordingly, the fitting effect between the original data and the predicted data under the three comparison methods is respectively listed in Figure 4, Figure 5 and Figure 6. The fitting effect diagram more intuitively reflects that the results under the latter two prediction methods have large errors.

**Figure 7:**
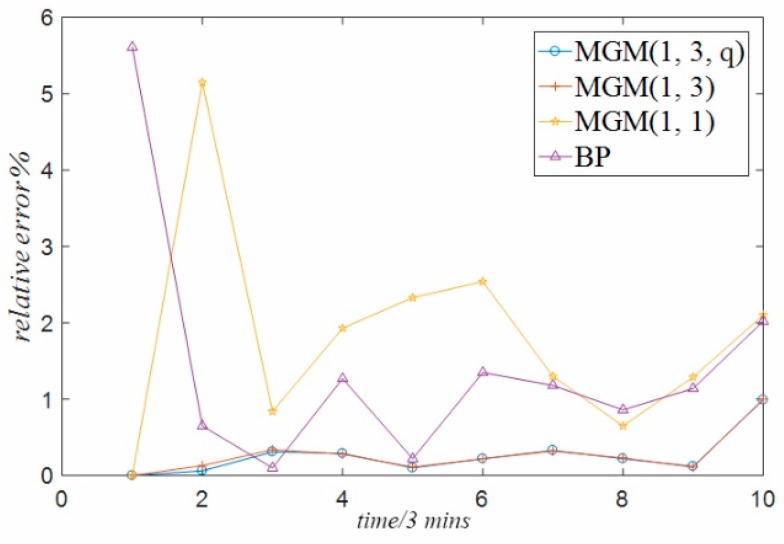
Relative error of guide bearing.

**Figure 8:**
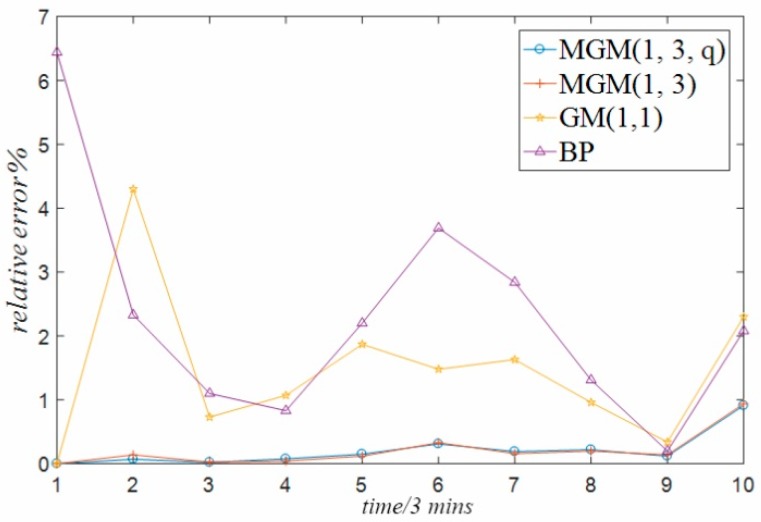
Relative error of stator winding.

**Figure 9:**
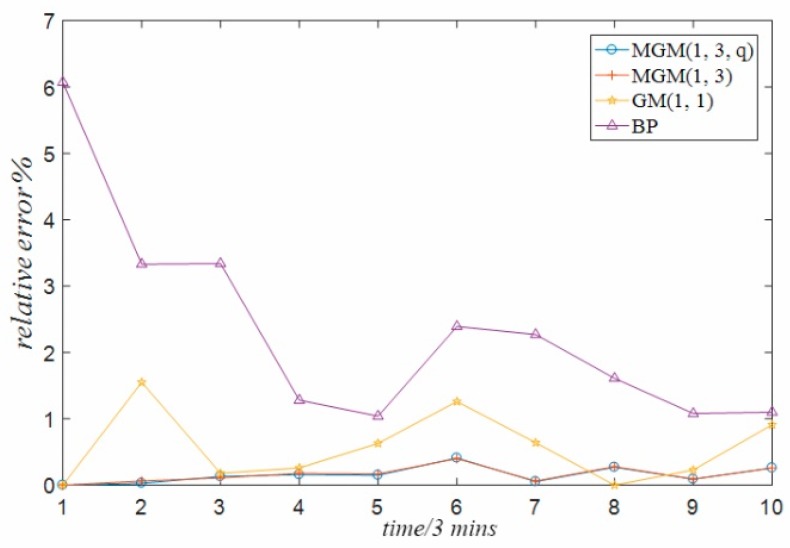
Relative error of thrust bearing.

The above comparative analysis compares and analyzes the prediction error of the data predicted by different forecasting methods. In order to more directly reflect the size of the prediction error generated by different forecasting methods, this paper further analyzes the errors generated by the prediction of temperature data of guide bearing, stator winding and temperature data of thrust bearing used in the experiment under the above four different methods, and forms a time series relative error graph, as shown in Figure 7, Figure 8 and Figure 9 respectively. It can be seen from the three graphs that, for each set of temperature data at different positions, there is always the minimum relative error of the prediction result of the multivariable grey model method optimized by particle swarm optimization proposed in this paper, while the prediction error of the single variable grey model method and BP neural network method is the largest. The reasons are analyzed in the following three aspects:

(1) The single-variable grey model only considers the influence of its own variables, but does not consider the coupling relationship between multiple variables. This is the defect relative to the multi-variable grey model method, which limits its prediction accuracy.
(2) Considering the practical application of the project, there is not enough temperature data in this paper, especially the temperature data in various modes to train the BP neural network model method. It is inevitable that the BP neural network model trained only with finite temperature data will have problems such as insufficient training and poor generalization due to over-fitting and combination. Therefore, the prediction accuracy of BP neural network model is low.
(3) The prediction accuracy of the general multi-variable grey model is high, but it is still lower than the optimized multi-variable grey model. This is because the default *q* parameter of the general multi-variable grey model is 0.5, which is not the optimal parameter.

## 6. Conclusions

MGM is used to process the original temperature data from multiple sensors of a pumping station unit and predict the changes of temperature data. It effectively overcomes the difficulties of the traditional time series method and the regression analysis method in dealing with non-stationary and nonlinear problems and overcomes the problem of the neural network method when the amount of data of the pumping station unit is small and cannot be accurately predicted.

PSO is used to optimize the *q* parameters in the MGM. The optimized MGM (1, *n*, *q*) is compared with traditional MGM (1, *n*), BP neural network method, and GM (1, 1).

Temperature, which is an important characteristic in evaluating the operation state of pumping station units, can be used to diagnose pumping station unit failures, helping predict when cracking will occur and exceed the safety threshold.

## Figures and Tables

**Table 1:** MGM (1, 3, *q*) model fitting value and error analysis.

No (*k*)	Real Sequence			MGM (1, 3, *q*) Prediction Sequence			Relative Error (%)		
	$x_{1}^{\left(0\right)}(k)$	$x_{2}^{\left(0\right)}(k)$	$x_{3}^{\left(0\right)}(k)$	${x^{\prime }}_{1}^{\left(0\right)}(k)$	${x^{\prime }}_{2}^{\left(0\right)}(k)$	${x^{\prime }}_{3}^{\left(0\right)}(k)$	$r_{1}^{\left(0\right)}(k)$	$r_{2}^{\left(0\right)}(k)$	$r_{3}^{\left(0\right)}(k)$
1	24.24	23.13	21.43	24.24	23.13	21.43	0	0	0
2	27.63	25.32	21.93	27.65	25.34	21.94	6.19 × 10^−2^	6.96 × 10^−2^	3.04 × 10^−2^
3	29.62	27.34	22.73	29.71	27.33	22.70	0.31	2.14 × 10^−4^	0.13
4	31.31	29.03	23.34	31.22	29.01	23.38	0.29	7.61 × 10^−4^	0.16
5	32.32	30.52	23.95	32.35	30.47	23.99	0.10	0.15	0.15
6	33.30	31.71	24.64	33.23	31.81	24.54	0.22	0.31	0.41
7	33.80	33.11	25.03	33.91	33.05	25.04	0.33	0.19	5.4 × 10^−2^
8	34.52	34.30	25.43	34.44	34.22	25.50	0.22	0.22	0.27
9	34.81	35.31	25.93	34.85	35.35	25.91	0.12	0.12	9.13 × 10^−2^
10	35.50	36.11	26.33	35.15	36.44	26.26	0.99	0.91	0.26
Mean relative error							0.26	0.19	0.15

**Table 2:** MGM (1, 3) model fitting value and error analysis.

No (*k*)	Real Sequence			MGM (1, 3, *q*) Prediction Sequence			Relative Error (%)		
	$x_{1}^{\left(0\right)}(k)$	$x_{2}^{\left(0\right)}(k)$	$x_{3}^{\left(0\right)}(k)$	${x^{\prime }}_{1}^{\left(0\right)}(k)$	${x^{\prime }}_{2}^{\left(0\right)}(k)$	${x^{\prime }}_{3}^{\left(0\right)}(k)$	$r_{1}^{\left(0\right)}(k)$	$r_{2}^{\left(0\right)}(k)$	$r_{3}^{\left(0\right)}(k)$
1	24.24	23.13	21.43	24.24	23.13	21.43	0	0	0
2	27.63	25.32	21.93	27.67	25.36	21.94	0.13	0.14	6.39 × 10^−2^
3	29.62	27.34	22.73	29.72	27.35	22.71	0.34	2.58 × 10^−2^	0.11
4	31.31	29.03	23.34	31.22	29.02	23.38	0.28	4.16 × 10^−2^	0.18
5	32.32	30.52	23.95	32.35	30.48	23.99	0.11	0.12	0.17
6	33.30	31.71	24.64	33.23	31.82	24.54	0.22	0.33	0.40
7	33.80	33.11	25.03	33.91	33.06	25.05	0.32	0.16	6.22 × 10^−2^
8	34.52	34.30	25.43	34.44	34.23	25.50	0.23	0.20	0.28
9	34.81	35.31	25.93	34.85	35.36	25.91	0.11	0.14	9.12 × 10^−4^
10	35.50	36.11	26.33	35.14	36.44	26.26	1.00	0.94	0.26
Mean relative error							0.27	0.21	0.15

**Table 3:** GM (1, 1) model fitting value and error analysis.

No (*k*)	Real Sequence			MGM (1, 3, *q*) Prediction Sequence			Relative Error (%)		
	$x_{1}^{\left(0\right)}(k)$	$x_{2}^{\left(0\right)}(k)$	$x_{3}^{\left(0\right)}(k)$	${x^{\prime }}_{1}^{\left(0\right)}(k)$	${x^{\prime }}_{2}^{\left(0\right)}(k)$	${x^{\prime }}_{3}^{\left(0\right)}(k)$	$r_{1}^{\left(0\right)}(k)$	$r_{2}^{\left(0\right)}(k)$	$r_{3}^{\left(0\right)}(k)$
1	24.24	23.13	21.43	24.24	23.13	21.43	0	0	0
2	27.63	25.32	21.93	29.05	26.41	22.27	5.15	4.30	1.55
3	29.62	27.34	22.73	29.87	27.54	22.77	0.84	0.73	0.18
4	31.31	29.03	23.34	30.71	28.72	23.28	1.93	1.07	0.26
5	32.32	30.52	23.95	31.57	29.95	23.8	2.33	1.87	0.63
6	33.30	31.71	24.64	32.45	31.24	24.33	2.54	1.48	1.26
7	33.80	33.11	25.03	33.36	32.57	24.87	1.30	1.63	0.64
8	34.52	34.30	25.43	34.30	33.97	25.43	0.65	0.96	0
9	34.81	35.31	25.93	35.26	35.43	25.99	1.29	0.34	0.23
10	35.50	36.11	26.33	36.25	36.94	26.57	2.10	2.30	0.91
Mean relative error							1.81	1.47	0.56

**Table 4:** Prediction value and error analysis of BP neural network model.

No (*k*)	Real Sequence			MGM (1, 3, *q*) Prediction Sequence			Relative Error (%)		
	$x_{1}^{\left(0\right)}(k)$	$x_{2}^{\left(0\right)}(k)$	$x_{3}^{\left(0\right)}(k)$	${x^{\prime }}_{1}^{\left(0\right)}(k)$	${x^{\prime }}_{2}^{\left(0\right)}(k)$	${x^{\prime }}_{3}^{\left(0\right)}(k)$	$r_{1}^{\left(0\right)}(k)$	$r_{2}^{\left(0\right)}(k)$	$r_{3}^{\left(0\right)}(k)$
1	24.24	23.13	21.43	25.60	24.62	20.13	5.61	6.44	6.07
2	27.63	25.32	21.93	27.45	25.91	21.20	0.65	2.33	3.33
3	29.62	27.34	22.73	29.65	27.64	21.97	0.10	1.10	3.34
4	31.31	29.03	23.34	31.71	28.79	23.04	1.27	0.83	1.28
5	32.32	30.52	23.95	32.25	29.85	23.70	0.22	2.20	1.04
6	33.30	31.71	24.64	33.75	30.54	24.05	1.35	3.69	2.39
7	33.80	33.11	25.03	34.20	32.17	24.46	1.18	2.84	2.27
8	34.52	34.30	25.43	34.82	33.85	25.02	0.86	1.31	1.61
9	34.81	35.31	25.93	35.21	35.24	25.65	1.14	0.20	1.08
10	35.50	36.11	26.33	36.22	36.86	26.04	2.02	2.08	1.10
Mean relative error							1.44	2.30	2.35
